# Supplementary material for: A fast Myosin super enhancer dictates muscle fiber phenotype through competitive interactions with Myosin genes
Source: Nat Commun. 2022 Feb 24;13:1039. doi: 10.1038/s41467-022-28666-1 (PMC8873246; doi:10.1038/s41467-022-28666-1)
Supplement: Supplementary file 1 — Supplementary Information [file 41467_2022_28666_MOESM1_ESM.pdf]

Supplementary Information

A fast *Myosin* super enhancer dictates muscle fiber phenotype through competitive interactions with *Myosin* genes

Matthieu Dos Santos et al

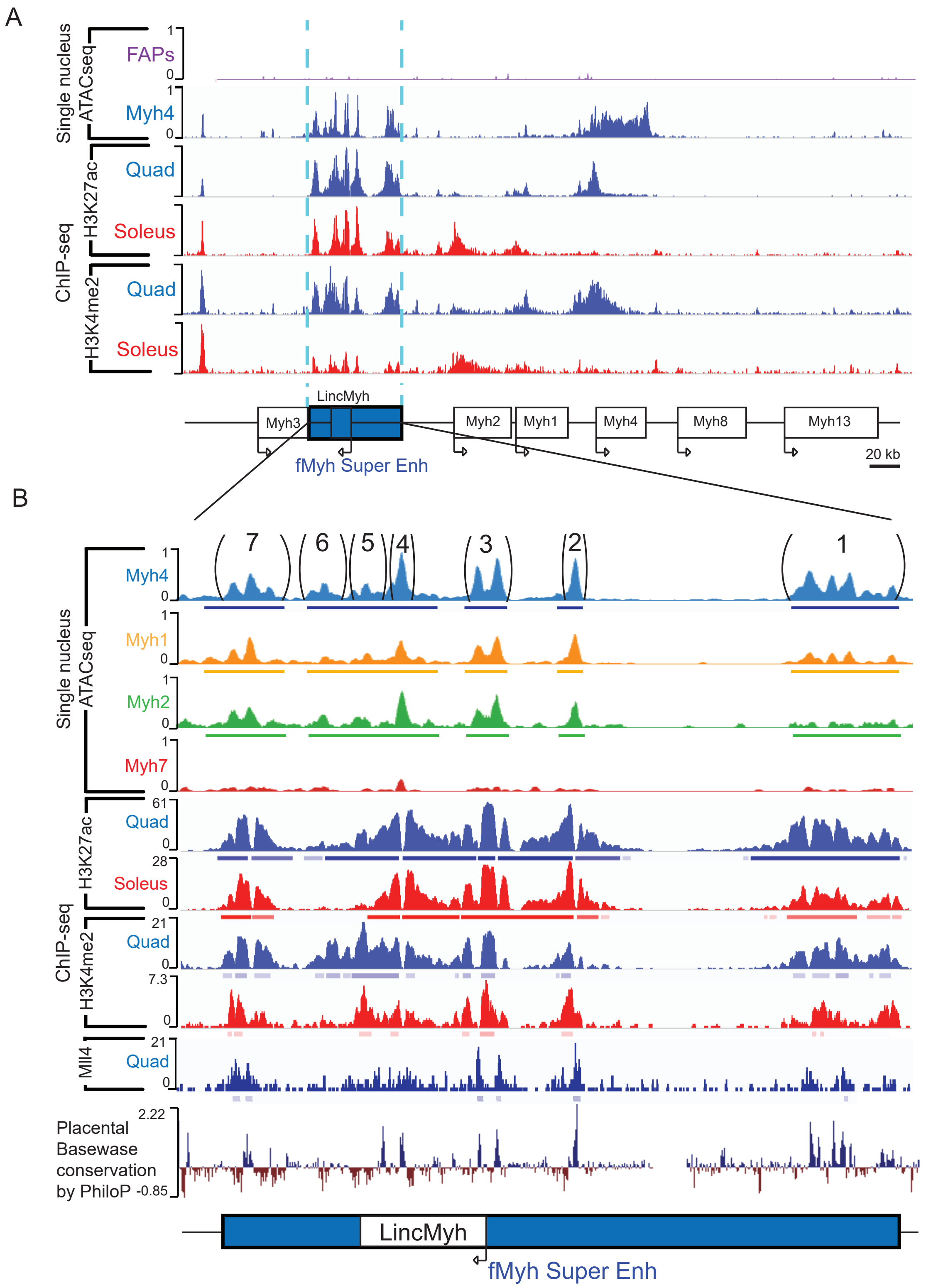

Supplementary Figure 1. Active histone marks in slow and fast muscles. (A) Chromatin accessibility as identified in snATAC-seq experiments in FAPs and *Myh4* myonuclei, in ChIP-seq experiments [1] for H3K27Ac and H3K4me2 in quadriceps and soleus in the f*Myh* locus. The fMyh locus is shown and the blue dashed blue lines align the snATAC-seq and

ChIP peaks with the locus, to scale. (B) Same as (A) with a zoom on the f*Myh* SE, and its seven peaks (in parenthesis, 1 to 7) as identified in *Myh4*+ myonuclei (blue lines under the peaks) in *Myh1*+ myonuclei (yellow lines under the peaks) and in *Myh2*+ myonuclei (green lines under the peaks) by snATAC-seq. ChIP-seq peaks for H3K27ac, H3K4me2 and Mll4 are also shown, from [1,2]. Below, Placental basewase conservation by PhiloP and multiple alignment of the DNA sequences from the 42kb SE are presented showing conserved regions (upper blue peaks) and less conserved regions (lower brown peaks). The position of the *Linc-Myh* gene inside the 42kb sequence is further illustrated. Ad.M.: adult skeletal muscles.

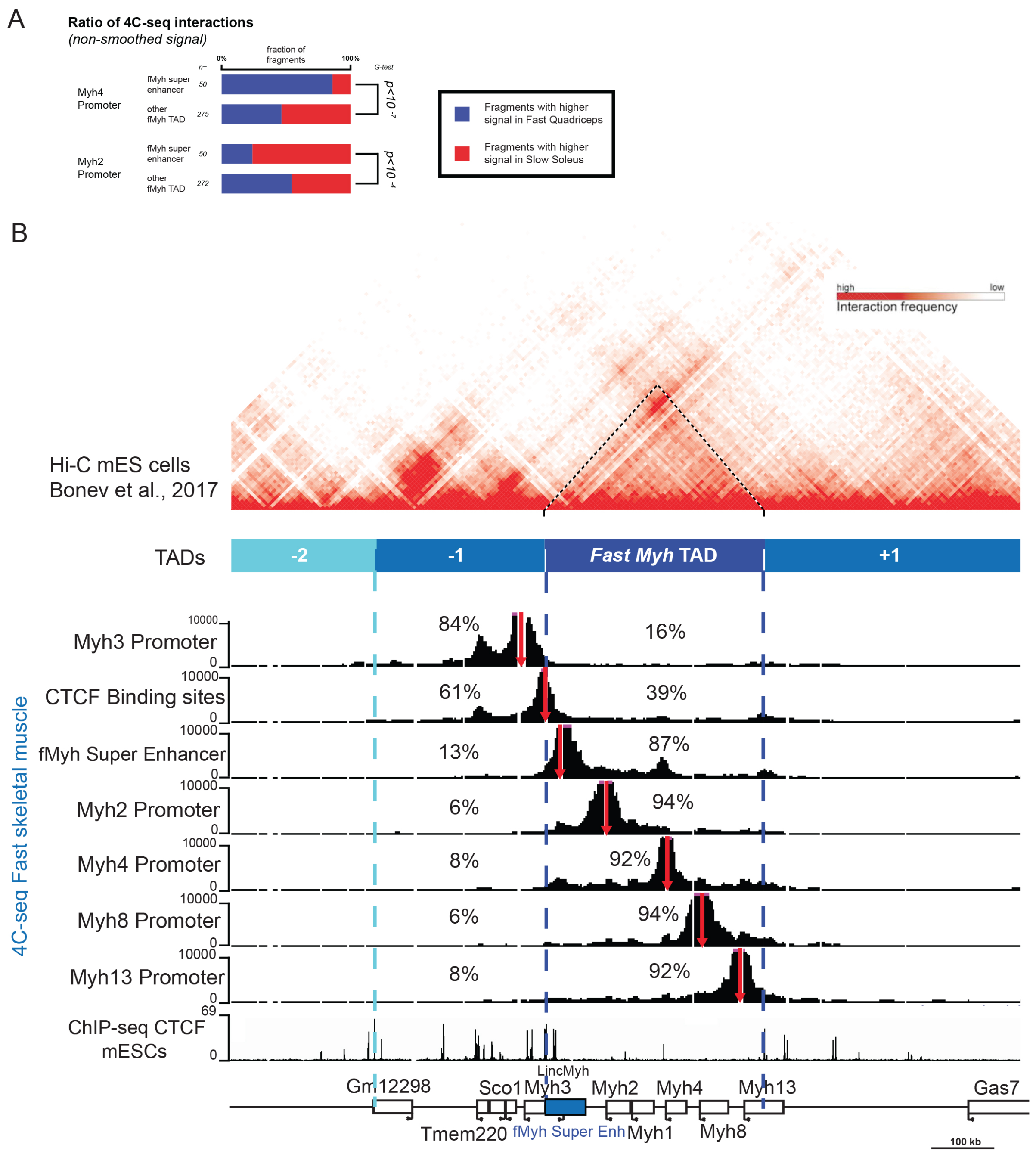

Supplementary Figure 2. Distinct TADs partitioned the f*Myh* locus as determined by HiC-seq experiments. (A) Distribution of 4C-seq signal for the *Myh4* (up) and *Myh2* (down) promoters with higher signal in quadriceps or soleus in the 42kb cis-regulatory f*Myh* SE or the other parts of the f*Myh* TAD. The total number of fragments in each region is indicated on the left. Significance of difference between the regions is indicated on the right, as calculated using a G-test of independence. (B) Alignment of Hi-C data from mES cells [3], 4C-seq data from adult fast skeletal muscles and ChIP-seq data against CTCF in mES cells [2] in the f*Myh* locus and adjacent TADs. HiC-seq data revealed that the f*Myh* locus is organized in one TAD delimited by CTCF borders at the 3' end of *Myh3* gene and at the 3' end of *Myh13*, showing that *Myh3* does not belong to the same TAD that the other f*Myh* genes. The size of the TAD

including *Myh1*, *Myh2* and *Myh4* is estimated at 350kb. For 4C-seq experiments, we used 7 distinct viewpoints in the f*Myh* locus to determine its organization in adult leg muscles. Viewpoints are indicated by red arrows.

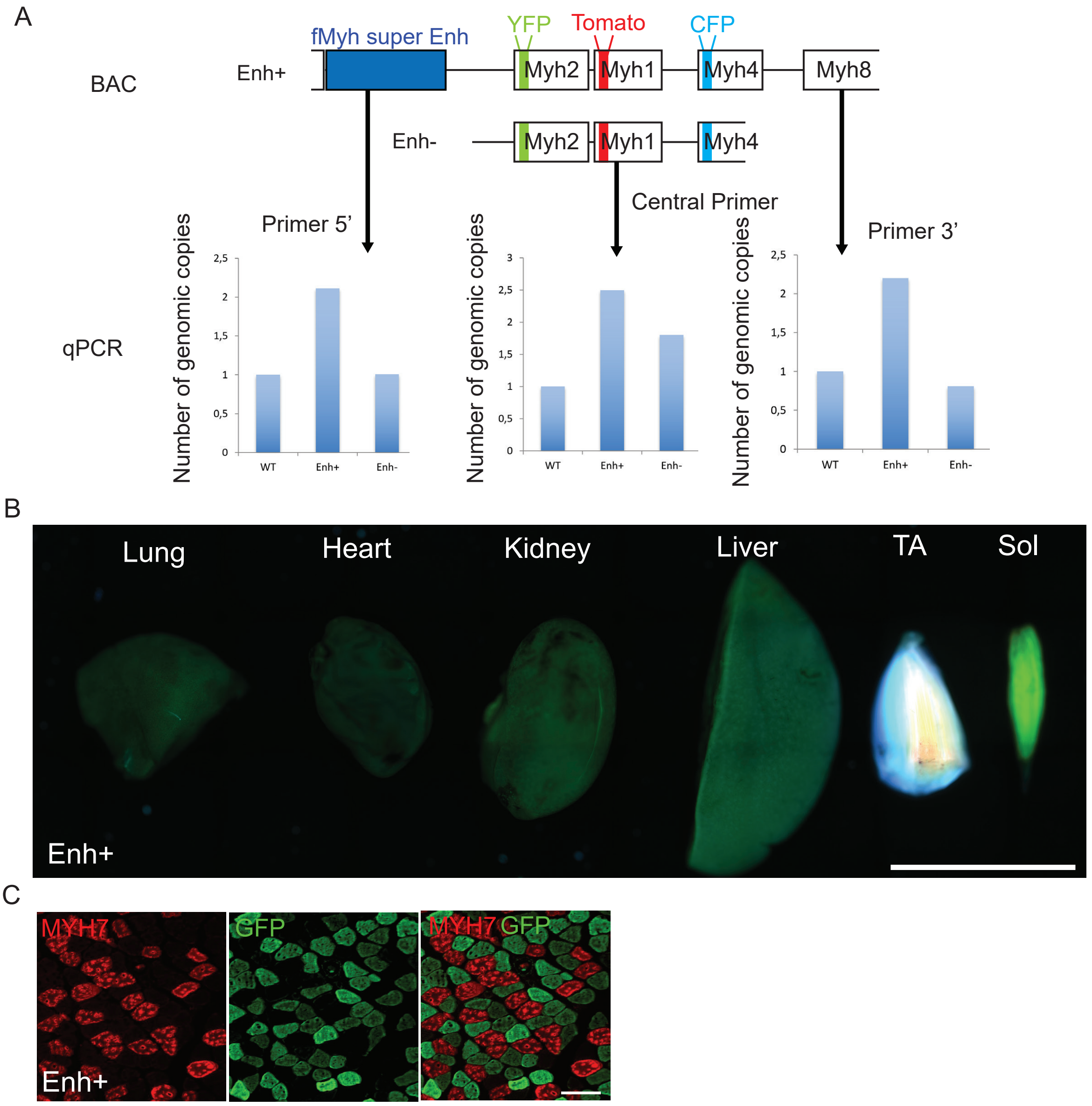

Supplementary Figure 3. f*Myh* regulation in Enh+ transgenic animals. (A) qPCR experiments to determine the copy number of integrated recombined BAC in WT, Enh+ and Enh- mouse genome. Several 5' and 3' primers were used to delineate the 5' and 3' deletion boundaries present in Enh- transgenic animals. For each line the experiments were done on the genomic DNA of six mice mixed together. The qPCRs were carried out by technical triplicates. (B) Transgenes expression in several tissues in Enh+ line. The expression of YFP, Tomato and CFP is only detected in skeletal muscle (TA and soleus in the picture) and not in other organs like lung, heart, kidney, or liver. (C) Immunostaining against endogenous MYH7 (red) and YFP (with an anti GFP antibody in green) on adult soleus of Enh + mice showing

that MYH7 fibers does not express YFP. Scale bars: 10mm for B and 100µm for F. Source data are provided as a Source Data file.

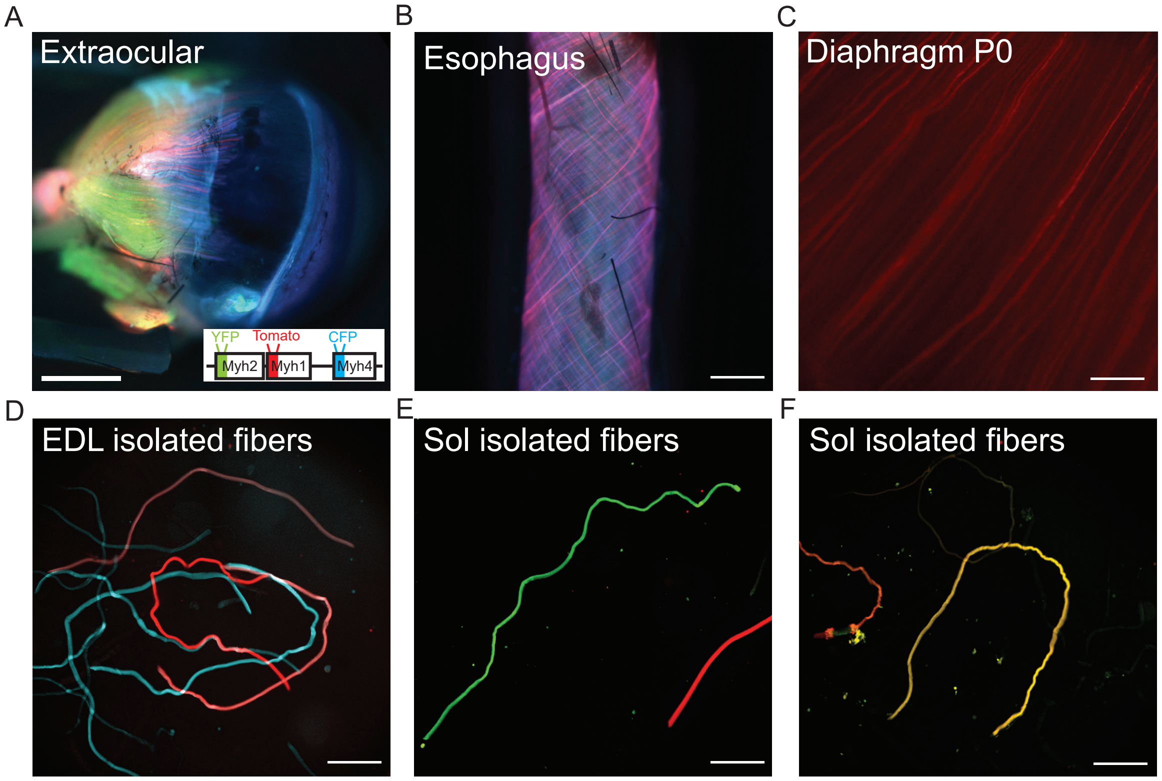

Supplementary Figure 4. Expression of the transgenes in skeletal muscles of Enh+ mice. (A) Schema of the three inserted transgenes in the f*Myh* BAC, and expression of the transgenes in extraocular muscles. Red; Tomato, green; YFP and blue; CFP. (B) same as in (A) showing the esophagus muscle of an adult Enh+ mouse. (C) Same as (A) showing the diaphragm in P0 Enh+ mouse. (D) Transgenes expression in isolated fibers from the EDL. We could detect pure YFP, Tomato and CFP fibers and the expression of the transgene is similar all along the fibers. At the top, a pink Tomato-CFP hybrid fiber. (E) The majority of soleus fibers express only one transgene; Tomato or YFP. (F) A minority of fibers co-expressed Tomato and YFP in adult soleus muscle. Scale bars: 1000µm for A, B, C, D, E and F.

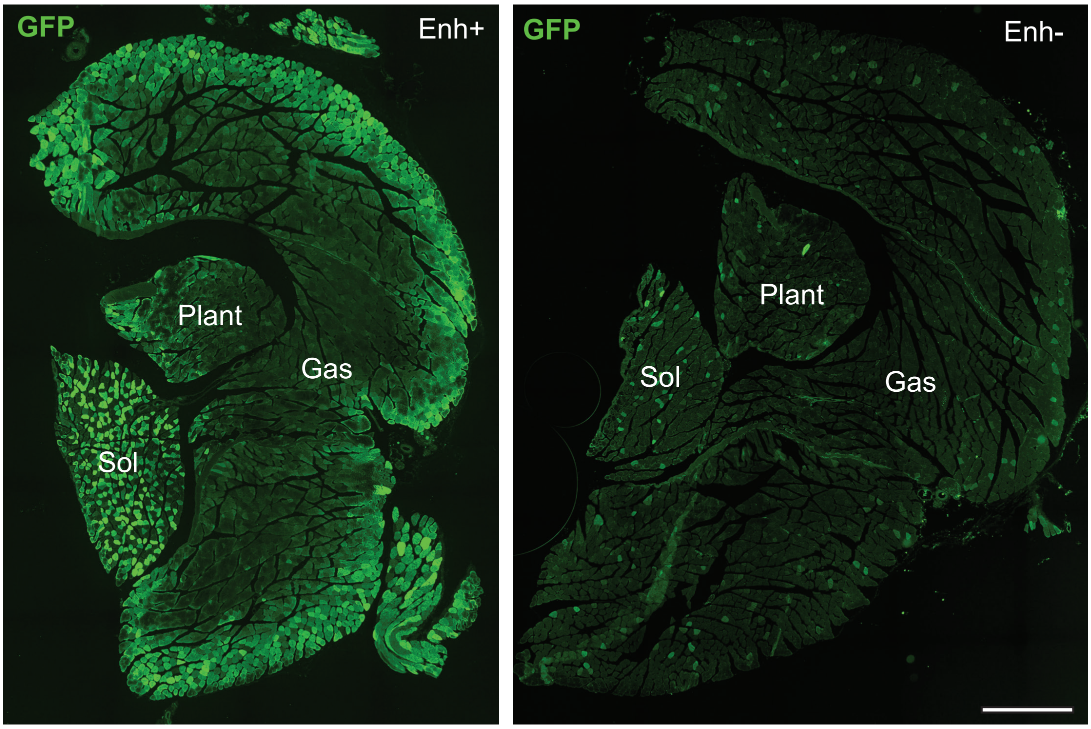

Supplementary Figure 5. Transgene expression in Enh- mouse. Immunostaining with GFP antibodies revealing YFP and CFP proteins on sections of adult soleus, plantaris and gastrocnemius in Enh+ (left) and Enh- (right) mice. Scale bar: 500µm.

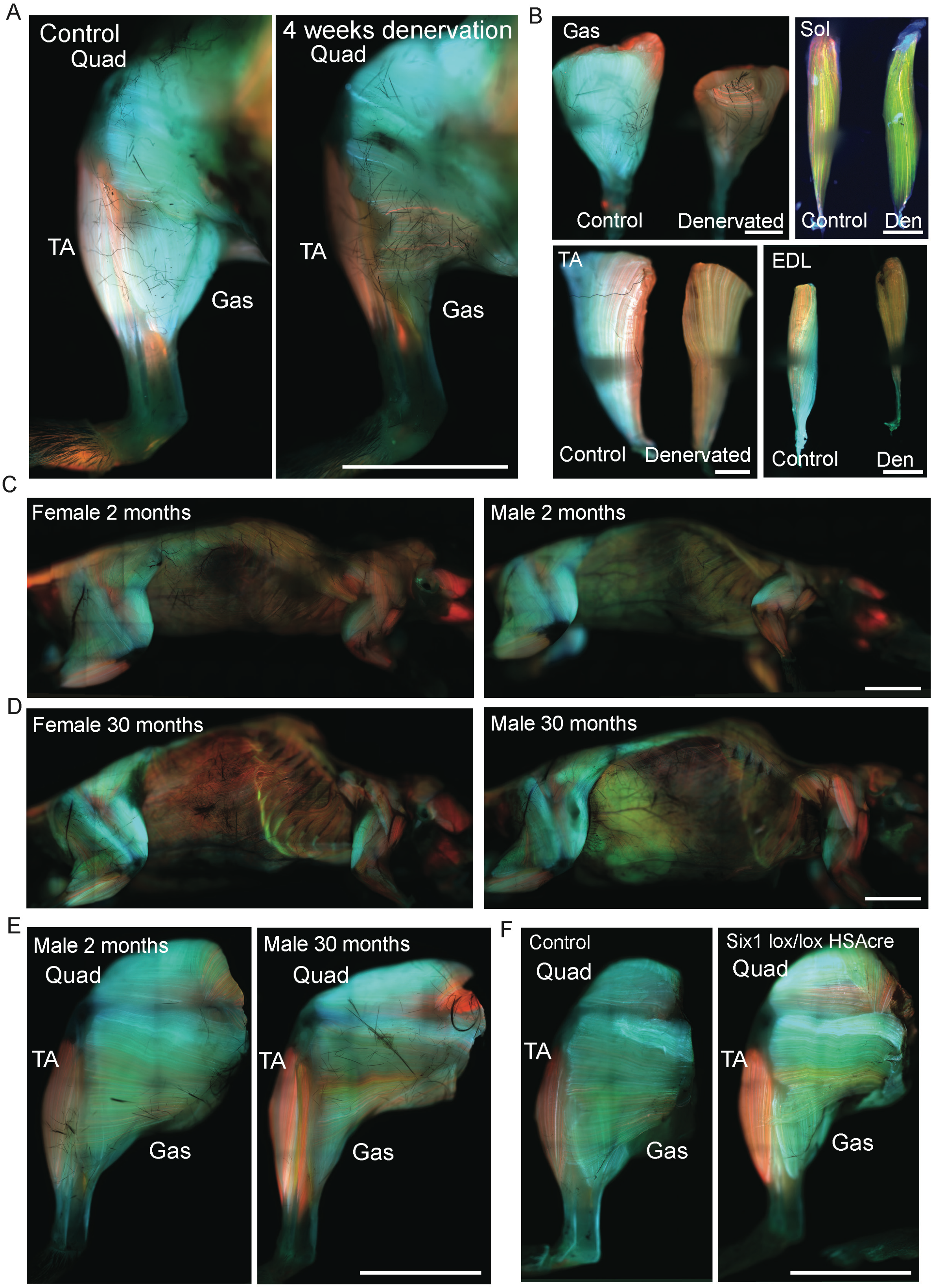

Supplementary Figure 6. Transgenic Enh+ mice allowed identification of fiber-type modifications under various pathophysiological conditions. (A) Control and 4 weeks denervated hindlimb legs of Enh+ mice. Distal muscles (TA, Gas) are more affected by the

absence of innervation than proximal muscles (Quad). A strong atrophy of the gastrocnemius is observed with a strong decrease of CFP expression. (B) Same as (A), zoom in Gastrocnemius, Tibialis anterior, EDL and soleus. Four weeks denervation induced a fast to slow transition in these 3 muscles visible by the expression of the transgenes. (C) 2-month-old Enh+ female and male mice. Skeletal muscles of females have more Tomato/MYH1 fibers and less CFP/MYH4 fibers than skeletal muscles of males. (D) Same as (C) in 30-month-old Enh+ female and male. (E) hindlimb of 2- and 30-month-old Enh+ mice. (F) 2 months old control and *Six1$^{flox/flox}$;HSA-CRE* hindlimb muscles of Enh+ mice. The absence of *Six1* in skeletal muscles induced a decreased number of CFP/MYH4 fibers and a fast to slow fiber type switch [4,5]. Gas: Gastrocnemius, TA: tibialis anterior, Sol: soleus. Scale bars: 6A: 10mm, 6B: 2mm, 6C: 10mm, 6D: 10mm, 6E: 10mm, 6F: 10mm.

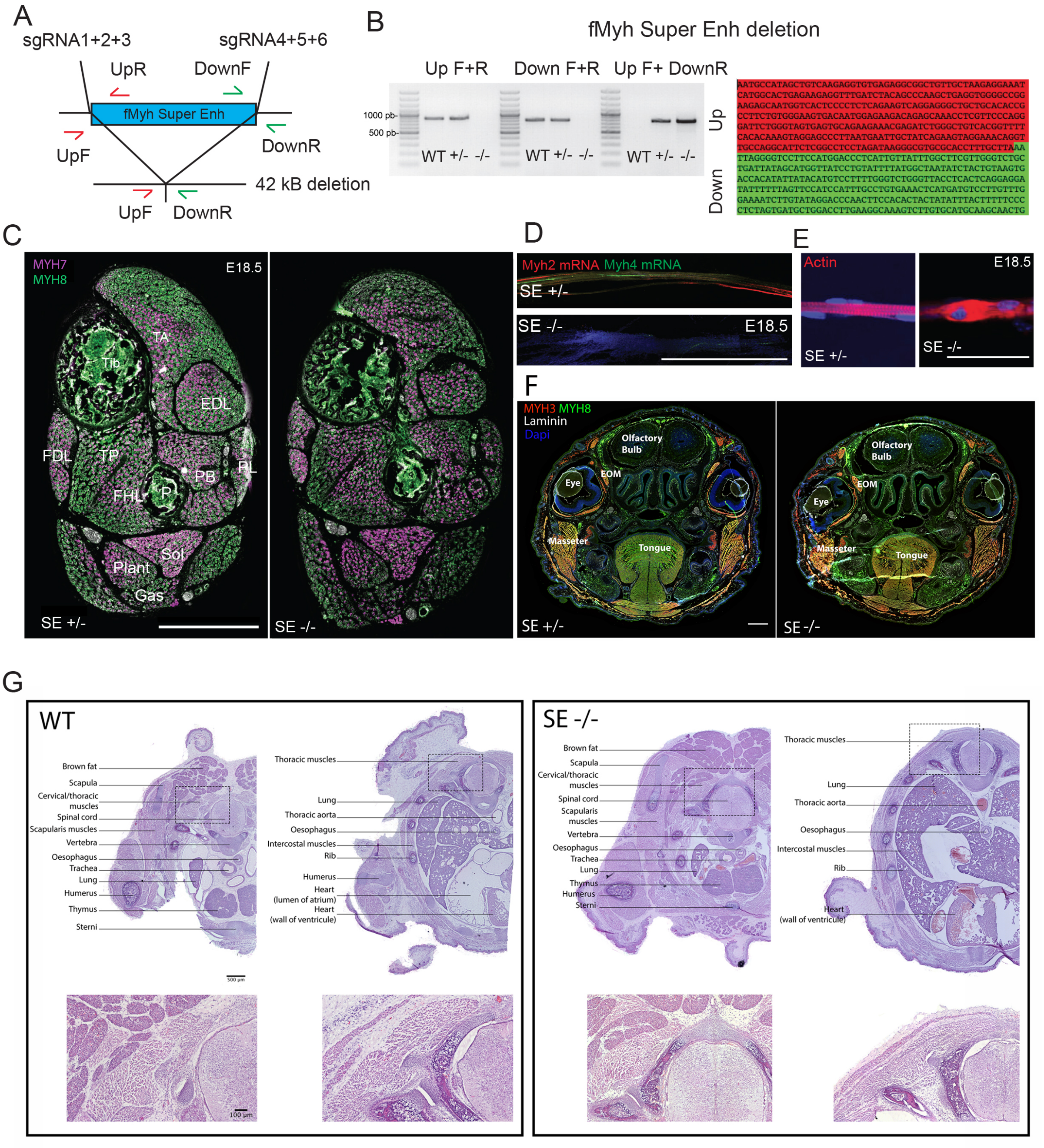

Supplementary Figure 7. Phenotype of f*Myh*-SE deletion in E18.5 mutant fetuses. (A) Diagram showing the strategy for *in vivo* deletion of the f*Myh-SE* by CRISPR/Cas9. (B) Sequence of the recombined allele in the region upstream of the up sgRNAs (Up in red) and downstream of down sgRNAs (Down in green) and demonstrating the deletion of the f*Myh* SE. (C) Immunostaining of distal hindlimb sections of control and *fMyh-SE*$^{-/-}$ showing MYH8 (green), MYH7 (purple) and Laminin (white) expression. (D) RNascope experiments against *Myh2* and *Myh4* mRNAs on isolated E18.5 fibers of control and mutant fetuses. (E) Myofibers from mutant diaphragm showed defects in sarcomeres organization as revealed by phalloidine staining. (F) Immunostaining of frontal head sections at the eye level of control

and f*Myh-SE*$^{-/-}$ showing MYH8 (green), MYH3 (red) Dapi (blue) and Laminin (white) expression. (G) Hematoxylin Eosin staining of transverse sections at the lung/heart levels in WT and f*Myh-SE*$^{-/-}$ fetuses. A zoom delimited by black doted square is presented below each picture. Scale bars: C: 100 µm, E: 500 µm, D:1mm. EOM: extra ocular muscles.

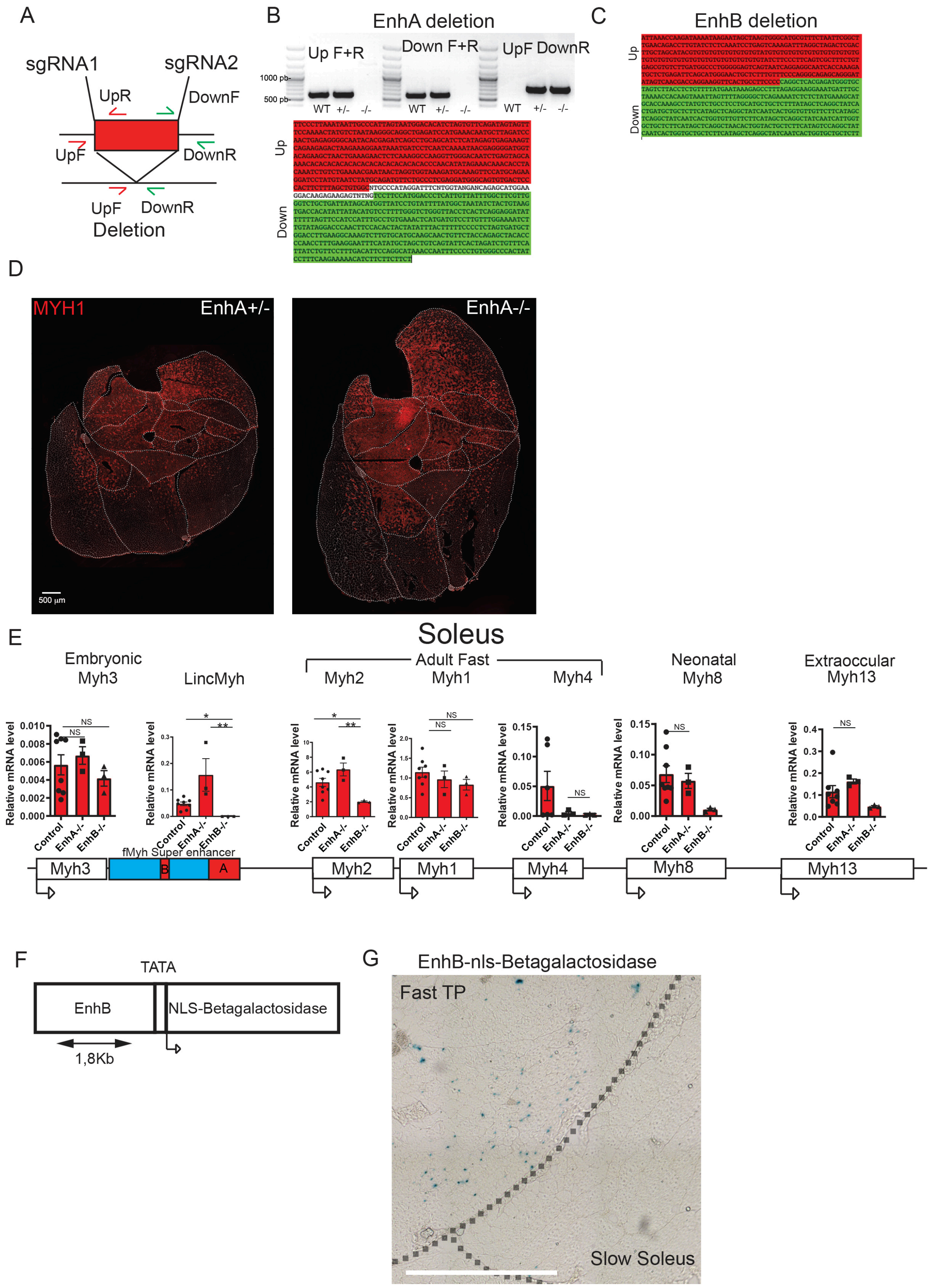

Supplementary Figure 8. Muscle phenotype of enhancers A and B deletion. (A) Diagram showing the strategies for *in vivo* deletions of enhancer A or B by CRISPR/Cas9. (B) PCR

showing the DNA fragment amplified after *EnhA* deletion, and sequence of the recombined allele in the region upstream of the up sgRNAs (Up in red) and downstream of down sgRNAs (Down in green) and demonstrating the deletion of the f*Myh EnhA*. (C) Sequence of the recombined allele in the region upstream of the up sgRNAs (Up in red) and downstream of down sgRNAs (Down in green) and demonstrating the deletion of the f*Myh EnhB*. (D) Immunostaining against MYH1 on adult distal hindlimb sections in control and $EnhA^{-/-}$. (E) Quantification of *Myh3, Myh2, Myh1, Myh4, Myh8* and *Myh13* mRNAs and of *Linc-Myh* by RT-qPCR in Soleus of control, *EnhA* and *EnhB* mutants. (F) Schema of the transgene used to generate a transgenic mouse line expressing nuclear beta-galactosidase under the control of the 1.8kb *EnhB* DNA element. (G) Beta-galactosidase positive nuclei are detected in the fast tibialis posterior but not in the slow soleus. For E, n=3. Numerical data are presented as mean ± S.E.M. $*P < 0.05$, $**P < 0.01$. Significance of difference, for Sup 8E: one way ANOVA with multiple comparisons. Scale bars: D and G: 500 µm. Source data are provided as a Source Data file.

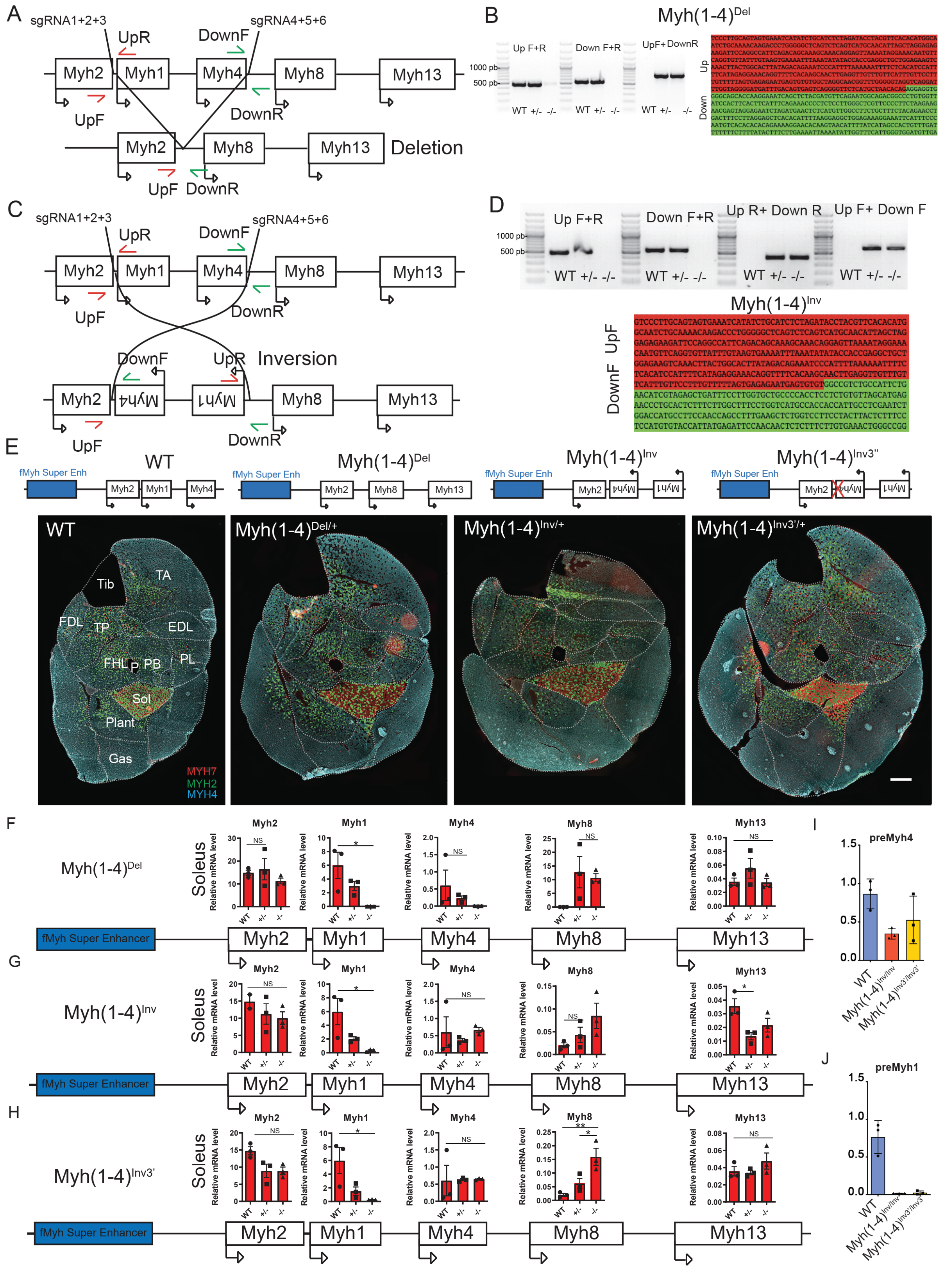

Supplementary Figure 9. Competition of the f*Myh* gene promoters for the shared SE. (A) Diagram showing the strategy for *in vivo* deletion of *Myh1* and *Myh4* by CRISPR/Cas9. (B) PCR used for the screening of mutant animals and sequence of the recombined allele in

the region upstream of the up sgRNAs (Up in red) and downstream of down sgRNAs (Down in green) and demonstrating the deletion of the *Myh1* and *Myh4* genes. (C) Diagram showing the strategy for *in vivo* inversion of *Myh1* and *Myh4* by CRISPR/Cas9. (D) PCR used for the screening of mutant animals and sequence of the recombined allele in the region upstream of the up sgRNAs (Up in red) and downstream of down sgRNAs (Down in green) and demonstrating the inversion of the *Myh1* and *Myh4* genes. (E) Immunostaining against MYH2, MYH4 and MYH7 proteins of adult distal hindlimb sections of 2-3-month-old WT, *Myh(1-4)*$^{Del/+}$, *Myh(1-4)*$^{Inv/+,}$ and of *Myh(1-4)*$^{Inv3'/+}$ animals. (F) Quantification of *Myh2, Myh1, Myh4, Myh8* and *Myh13* mRNAs by RT-qPCR from adult Soleus of WT, *Myh(1-4)*$^{Del/+}$, and *Myh(1-4)*$^{Del/Del}$ animals, (n=3). (G) Quantification of *Myh2, Myh1, Myh4, Myh8* and *Myh13* mRNAs by RT-qPCR from adult Soleus of WT, *Myh(1-4)*$^{Inv/+}$ and *Myh(1-4)*$^{Inv/Inv}$ animals, (n=3). (H) Quantification of *Myh2, Myh1, Myh4, Myh8* and *Myh13* mRNAs by RT-qPCR from adult Soleus of WT, *Myh(1-4)*$^{Inv3'/+}$ and of *Myh(1-4)*$^{Inv3'/Inv3'}$ animals, (n=3). (I) Quantification of *Myh4* premRNA from adult TA of WT, *Myh(1-4)*$^{Inv/Inv}$ and of *Myh(1-4)*$^{Inv3'/Inv3'}$ animals, (n=3). (J) Quantification of *Myh1* premRNA from adult TA of WT, *Myh(1-4)*$^{Inv/Inv}$ and of *Myh(1-4)*$^{Inv3'/Inv3'}$ animals, (n=3). Numerical data are presented as mean ± S.E.M. $*P < 0.05$, $**P < 0.01$. Significance of difference, for Sup 9F, Sup 9G and Sup 9H: one way ANOVA with multiple comparisons. For E, scale bar: 500 µm. Source data are provided as a Source Data file.

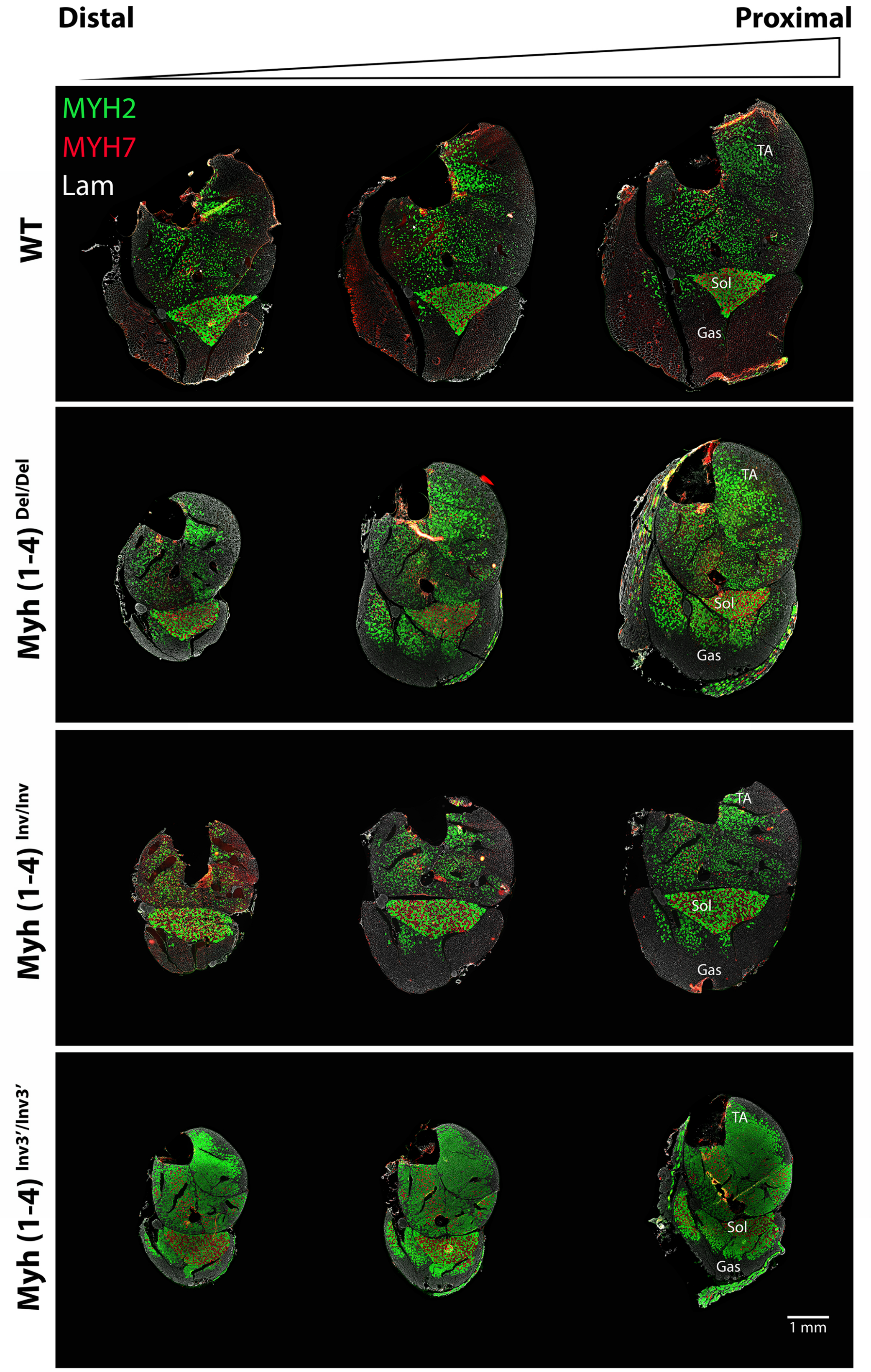

Supplementary Figure 10. Expression of f*Myh* in *Myh(1-4)*^{Del}, *Myh(1-4)*^{Inv,} and *Myh(1-4)*$^{Inv3'}$ adult mice. Immunostaining against fast MYH2, slow MYH7 and Laminin on adult leg sections along the proximo-distal axis of distal hindlimb in WT, *Myh(1-4)*$^{Del/Del}$ *Myh(1-4)*$^{Inv/Inv}$ and *Myh(1-4)*$^{Inv3'/Inv3'}$ of 2-3-month-old mouse female. The TA, Sol and Gas are indicated. Scale bar: 1mm.

1 Ramachandran, K. *et al.* Dynamic enhancers control skeletal muscle identity and reprogramming. *PLoS Biol* **17**, e3000467, doi:10.1371/journal.pbio.3000467 (2019).

2 Liu, L. *et al.* Histone methyltransferase MLL4 controls myofiber identity and muscle performance through MEF2 interaction. *J Clin Invest* **130**, 4710-4725, doi:10.1172/JCI136155 (2020).

3 Bonev, B. *et al.* Multiscale 3D Genome Rewiring during Mouse Neural Development. *Cell* **171**, 557-572 e524, doi:10.1016/j.cell.2017.09.043 (2017).

4 Sakakibara, I. *et al.* Six1 homeoprotein drives myofiber type IIA specialization in soleus muscle. *Skelet Muscle* **6**, 30, doi:10.1186/s13395-016-0102-x (2016).

5 Maire, P. *et al.* Myogenesis control by SIX transcriptional complexes. *Semin Cell Dev Biol* **104**, 51-64, doi:10.1016/j.semcdb.2020.03.003 (2020).

Primary antibodies

Target	Species	Antibody reference	Supplier	dilution
Myh7	mouse IgG2B	BA-F8	DHSB	1/40
Myh2	mouse IgG1	SC-71	DHSB	1/200
Myh1	mouse IgM	6H1	DHSB	1/40
Myh4	mouse IgM	BF-F3	DHSB	1/200
Myh8	mouse IgG2a	N3.36	DHSB	1/200
Myh13	mouse IgM	4A6	DHSB	1/200
Myh3	mouse IgG1	BF-45	DHSB	1/200
Laminin	Rabbit	L9393	Sigma	1/500
GFP	Chicken	ab290	Abcam	1/200

Secondary antibodies

Target	Wave length (nm)	Antibody reference	Supplier	dilution
Goat anti mouse IgG2b	350	A21140	Invitrogen	1/500
Goat anti mouse IgG2a	546	A21133	Invitrogen	1/1000
Goat anti mouse IgG1	546	A21123	Invitrogen	1/1000
Goat anti mouse IgM	647	A21238	Invitrogen	1/1000
Goat anti rabbit IgG	488	A11008	Invitrogen	1/1000
Goat anti chicken IgG	488	A11039	Invitrogen	1/1000

Toxin

Target	Conjugated Toxin	Reference	Supplier	dilution
AChR	alpha bungarotoxin-488	B13422	Thermofischer	1/500

Supplementary Table 1. Antibodies used in the study.

	Forward	reverse
Myh7	CTCAAGCTGCTCAGCAATCTATTT	GGAGCGCAAGTTTGTCATAAGT
Myh2	CGGGAGTCTTGGTTTCATTG	CCAAGAAAGGTGCCAAGAAG
Myh1	CAGGAGTCTTGGTTTCATT	CGGTGGTGGAAAGAAAGG
Myh4	GCTTGAAAACGAGGTGGAAA	CCTCCTCAGCCTGTCTCTTG
Myh8	CAGGAGCAGGAATGATGCTCTGAG	AGTTCCTCAAACTTTCAGCAGCCAA
Myh13	GAAGCTCCTGAACTCCATCG	GGTCACCAGCTTCTCGTCTC
LincMyh	GTGCAGCCAGAACAAGACAG	CAAGATGGGAGGCTCTCAAA
YFP	TTTCCCAGCTGCACCTTCTC	GCTGAACTTGTGGCCGTTTA
Tomato	AGTTCATGCGCTTCAAGGTG	TGGAGCCGTACATGAACTGG
CFP	TTGTCTGACTCAAGCCTGCC	CCGGTGGTGCAGATGAACTT

Supplementary Table 2. Oligonucleotides used for RT-qPCR experiments.

4C_My3_s2_iF1+adap		AATGATACGGCGACCACCGAGATCTACACTCTTTCCCTACACGACGCTCTTCCGATC**AGGGGCTCCTAAAGCAAAGGA**
4C_My3_s2_iR1		CAAGCAGAAGACGGCATACGAGATCGTGATGTGACTGGAGTTCAGACGTGTGCTCTTCCGATCT**ACAGGGAGCTATGCCACCAG**
4C_CTCF_s1_iF1		AATGATACGGCGACCACCGAGATCTACACTCTTTCCCTACACGACGCTCTTCCGATC**GAGAATCTCTGACTGGGAAATA**
4C_CTCF_s1_iR1		CAAGCAGAAGACGGCATACGAGATCGTGATGTGACTGGAGTTCAGACGTGTGCTCTTCCGATCT**TTCTCTCTAGATGGTGGTCC**
4C_Mf6_s2_iF1		AATGATACGGCGACCACCGAGATCTACACTCTTTCCCTACACGACGCTCTTCCGATC**CAATCGCAGCCCCTTGCT**
4C_Mf6_s2_iR1		CAAGCAGAAGACGGCATACGAGATCGTGATGTGACTGGAGTTCAGACGTGTGCTCTTCCGATCT**ACCCAAGAGGTGAGTGACAGT**
4C_DHS_s1_iF1		AATGATACGGCGACCACCGAGATCTACACTCTTTCCCTACACGACGCTCTTCCGATC**CAGAACTGAACACCACCC**
4C_DHS_s1_iR1		CAAGCAGAAGACGGCATACGAGATCGTGATGTGACTGGAGTTCAGACGTGTGCTCTTCCGATCT**GGCAGAACTGAACACCAC**
4C_MYH2_s1_iF1		AATGATACGGCGACCACCGAGATCTACACTCTTTCCCTACACGACGCTCTTCCGATC**TCTCCTATGGATAATTTGAGAAGAT**
4C_MYH2_s1_iR1		CAAGCAGAAGACGGCATACGAGATCGTGATGTGACTGGAGTTCAGACGTGTGCTCTTCCGATCT**GGAGGCGTGATGGAGAAT**
4C_MYH4_s2_iF1		AATGATACGGCGACCACCGAGATCTACACTCTTTCCCTACACGACGCTCTTCCGATC**AACTATGAAGGCTGGGCT**
4C_MYH4_s2_iR1		CAAGCAGAAGACGGCATACGAGATCGTGATGTGACTGGAGTTCAGACGTGTGCTCTTCCGATCT**AGTGGAAGAGGAGCAGTC**
4C_MYH1_s1_2_iF1		AATGATACGGCGACCACCGAGATCTACACTCTTTCCCTACACGACGCTCTTCCGATC**AGTGTCACCATCCAGAGT**
4C_MYH1_s1_2_iR1		CAAGCAGAAGACGGCATACGAGATCGTGATGTGACTGGAGTTCAGACGTGTGCTCTTCCGATCT**TAAGAGACTTAAGCATTGATAGGT**
4C_MYH13_s3_iF1		AATGATACGGCGACCACCGAGATCTACACTCTTTCCCTACACGACGCTCTTCCGATC**GAGGAAGTGAGCTGTTACG**
4C_MYH13_s3_iR1		CAAGCAGAAGACGGCATACGAGATCGTGATGTGACTGGAGTTCAGACGTGTGCTCTTCCGATCT**TGTTCTACAATGCACCCC**

Supplementary Table 3. Oligonucleotides used for 4C-seq experiments.

Super Enhancer deletion		
sgRNA Up1	GGGGACTCCTATGTAAGCAA	
sgRNA Up2	TCTAGGAGGCCGAGAATGCC	
sgRNA Up3	TTGGGAGCAGTCCCATCTGG	
sgRNA Down1	CCATGGAAGGACCCCTAATT	
sgRNA Down2	CAGACAAGGCCTTCAATTAG	
sgRNA Down3	TCTCTGACATCAAGCCATGC	
PCR primer	forward	reverse
super_enh_Up	TTGCGGCTATGGAAGGAAGG	GAGTTCAGTCCCCAAGGCAA
super_enh_down	GGGTTCCTTGCCTCTGTACC	TCCAGCATCACTAGAGGGGA
EnhA deletion		
sgRNA Up1	GAGAAGAGTCTCGTCATTGTGG	
sgRNA Up2	GTCACACTGCCCATCCTCGAGGG	
sgRNA Up3	GATGTTCTGCCCTCGAGGATGGG	
sgRNA Down1	GATGGAAGGACCCCTAATTAGG	
sgRNA Down2	GCAGACAAGGCCTTCAATTAGGGG	
sgRNA Down3	GTCTCTGACATCAAGCCATGCAGG	
PCR primer	forward	reverse
EnhA_Up	CACACACACACACACCCAAC	TTCCCATCACCTGAGTCTCC
EnhA_down	CCCCATTTCACCTTCAGAGA	CCTTCAAGGTCCAGCATCAC
EnhB deletion		
sgRNA Up1	GTGACTATCCAGAAGTCTGG	
sgRNA Down1	CCTTTCAGGCTCACGAGATGG	
PCR primer	forward	reverse
EnhB_Up	GCTAAGTGGGCATGCGTTTC	CAAAGCAGGACCCAAACAGC
EnhB_down	TCCCGTTCCTTGATCCCTCT	GGCTTTGGTGCATGCTTTCA
Myh1-4 deletion and inversion		
sgRNA Up1	ATGAGTGTGTGGCTAGGCAACGG	
sgRNA Up2	GTGGCTAGGCAACGGTTTGGGGG	
sgRNA Up3	ATGATTTGACAGTGAGTCAGAGG	
sgRNA Down1	GGGTTCTCATGCTAACACAGAGG	
sgRNA Down2	AAGTGAAGTGGATAACCACAGGG	
sgRNA Down3	CAGAATGGCAGACGGCCCTGTGG	
PCR primer	forward	reverse
Myh1_Up	GGCCATTCAGACAGCAAAGC	GCTCCTCAGTGTAGCTGCAA
Myh4_down	GGACCAGAGCTTCAAAGGCT	CTCAAAGTGGCCTTCCCGAT

Supplementary Table 4. sgRNA and PCR primers used for screening of CRISPR/Cas9 edited genome.
