# Supplementary material for: A fast Myosin super enhancer dictates muscle fiber phenotype through competitive interactions with Myosin genes
Source: Nat Commun. 2022 Feb 24;13:1039. doi: 10.1038/s41467-022-28666-1 (PMC8873246; doi:10.1038/s41467-022-28666-1)
Supplement: Supplementary file 3 — Reporting Summary [file 41467_2022_28666_MOESM3_ESM.pdf]

nature portfolio

Corresponding author(s): Pascal Maire

Last updated by author(s): Jan 25, 2022

Reporting Summary

Nature Portfolio wishes to improve the reproducibility of the work that we publish. This form provides structure for consistency and transparency in reporting. For further information on Nature Portfolio policies, see our Editorial Policies and the Editorial Policy Checklist.

Statistics

For all statistical analyses, confirm that the following items are present in the figure legend, table legend, main text, or Methods section.

n/a	Confirmed	
☐	☒	The exact sample size (*n*) for each experimental group/condition, given as a discrete number and unit of measurement
☐	☒	A statement on whether measurements were taken from distinct samples or whether the same sample was measured repeatedly
☐	☒	The statistical test(s) used AND whether they are one- or two-sided<br>*Only common tests should be described solely by name; describe more complex techniques in the Methods section.*
☐	☒	A description of all covariates tested
☒	☐	A description of any assumptions or corrections, such as tests of normality and adjustment for multiple comparisons
☐	☒	A full description of the statistical parameters including central tendency (e.g. means) or other basic estimates (e.g. regression coefficient) AND variation (e.g. standard deviation) or associated estimates of uncertainty (e.g. confidence intervals)
☐	☒	For null hypothesis testing, the test statistic (e.g. *F*, *t*, *r*) with confidence intervals, effect sizes, degrees of freedom and *P* value noted<br>*Give P values as exact values whenever suitable.*
☒	☐	For Bayesian analysis, information on the choice of priors and Markov chain Monte Carlo settings
☒	☐	For hierarchical and complex designs, identification of the appropriate level for tests and full reporting of outcomes
☒	☐	Estimates of effect sizes (e.g. Cohen's *d*, Pearson's *r*), indicating how they were calculated

Our web collection on statistics for biologists contains articles on many of the points above.

Software and code

Policy information about availability of computer code

Data collection	Metamorph 7.7.5
Data analysis	Cell Ranger Single Cell Software suite 3.0.2, R(V3.4.3), Seurat (V3.0.2), Signac (V0.2.4), Fiji (V2.0.0), Graphpad (V6.0)

For manuscripts utilizing custom algorithms or software that are central to the research but not yet described in published literature, software must be made available to editors and reviewers. We strongly encourage code deposition in a community repository (e.g. GitHub). See the Nature Portfolio guidelines for submitting code & software for further information.

Data

Policy information about availability of data

All manuscripts must include a data availability statement. This statement should provide the following information, where applicable:

- Accession codes, unique identifiers, or web links for publicly available datasets
- A description of any restrictions on data availability
- For clinical datasets or third party data, please ensure that the statement adheres to our policy

Single nucleus ATAC-seq data are available in the NCBI Gene Expression Omnibus (GEO) database under accession GSE150065 and 4C-seq data GSE168074

Field-specific reporting

Life sciences study design

All studies must disclose on these points even when the disclosure is negative.

Sample size	SnATAC-seq experiments have been published already (Dos Santos et al, 2020). For4C-seq experiments, we used more than ten animals per condition.This number of animals is sufficient to obtain statistically significant differences. Significant differences between mean values were evaluated using two-way ANOVA or t-Student tests with Graphpad 6 software.
Data exclusions	We excluded no data.
Replication	For all experiments, three biological replicates were used for each condition, giving rise to the same results.
Randomization	Experiments have been performed randomly. Samples were allocated into experimental groups randomly depending on the condition.
Blinding	The investigators were not blinded in regard to allocation of samples during experiments and outcome assessment because of technical limitation and impossibility of blinding the investigators.<br>Moreover, the blinding is used mainly in the case of clinical studies where the number and the variations between samples are high.<br>In our study (fundamental research), we use inbred mouse lines reducing the interindividual variability. The different samples were treated as equally as possible.

Reporting for specific materials, systems and methods

We require information from authors about some types of materials, experimental systems and methods used in many studies. Here, indicate whether each material, system or method listed is relevant to your study. If you are not sure if a list item applies to your research, read the appropriate section before selecting a response.

Materials & experimental systems

n/a	Involved in the study	
☐	☒	Antibodies
☒	☐	Eukaryotic cell lines
☒	☐	Palaeontology and archaeology
☐	☒	Animals and other organisms
☒	☐	Human research participants
☒	☐	Clinical data
☒	☐	Dual use research of concern

Methods

n/a	Involved in the study	
☒	☐	ChIP-seq
☒	☐	Flow cytometry
☒	☐	MRI-based neuroimaging

Antibodies

Antibodies used

Target Antibody reference Supplier Species dilution
Myh7 BA-F8 DHSB mouse IgG2B 1/40.
Myh2 SC-71 DHSB mouse IgG1 1/200
Myh1 6H1 DHSB mouse IgM 1/40.
Myh4 BF-F3 DHSB mouse IgM 1/200
Laminin L9393 Sigma Rabbit 1/500
Myh3 BF-45 DHSB mouse IgG1 1/200
Myh8 N3.36 DHSB mouse IgG2a 1/200
Myh13 4A6 DHSB mouse IgM 1/200
GFP ab290 Abcam Chicken 1/200

Goat anti mouse IgG2b-350 A21140 Invitrogen, 1/500
Goat anti mouse IgG1-546 A21123 Invitrogen , 1/1000
Goat anti mouse IgM-647 A21238 Invitrogen, 1/1000
Goat anti rabbit IgG-488 A11008 Invitrogen, 1/1000
Goat anti chicken IgG-488 A11039 Invitrogen, 1/1000

Validation

The antibodies have been validated by several studies as specified by:
PMID: 22530000 for Myh antibodies
PMID: 29733324 for Laminin antibodies
For GFP antibodies ab290 Abcam, PMID: 32123325

Animals and other organisms

Policy information about studies involving animals; ARRIVE guidelines recommended for reporting animal research

Laboratory animals	6-8 weeks old C57bl6N females were used in this study. Mice were maintained at temperature 22+/-2 °C, with 30 to 70% humidity and with a dark/light cycle of 12h/12h.
Wild animals	No wild animals were used.
Field-collected samples	No field collected samples.
Ethics oversight	Animal experimentations were carried out in strict accordance with the European STE123 and the French national charter of Ethics of Animal Experimentation. Protocols were approved by the Ethical Committee of Animal experiments of the Institut Cochin, CNRS UMR 8104, INSERM U1016, and by the Ministere de l'Education nationale, de l'enseignement superieur et de la recherche, n° APAFIS #15699-2018021516569195.

Note that full information on the approval of the study protocol must also be provided in the manuscript.
